# Precancerous Gallbladder Lesions in Cholelithiasis: A Histopathological Study

**DOI:** 10.7759/cureus.99139

**Published:** 2025-12-13

**Authors:** Umesh Choudhary, Sanjay Kumar Saroj, Devendra Singh Shekhawat, Md Jawed Akhtar, Satyanam Kumar Bhartiya, Anand Mishra

**Affiliations:** 1 Department of Anatomy, Institute of Medical Sciences, Banaras Hindu University, Varanasi, IND; 2 Department of General Surgery, Institute of Medical Sciences, Banaras Hindu University, Varanasi, IND; 3 Department of Anatomy, Indira Gandhi Institute of Medical Sciences, Patna, IND

**Keywords:** alkaline phosphatase, cholelithiasis, dysplasia, gallbladder cancer, hyperplasia, metaplasia

## Abstract

Background: Gallbladder cancer (GBC) is highly prevalent in Northern and Eastern India and often diagnosed late, leading to poor outcomes. Identifying precursor lesions is essential, as the metaplasia-dysplasia-carcinoma sequence plays a central role in gallbladder carcinogenesis. This study examines precancerous mucosal changes in cholelithiasis and evaluates their histopathological patterns and clinical relevance for early detection.

Materials and methods: This cross-sectional study included 100 patients with ultrasonography-confirmed cholelithiasis who underwent cholecystectomy at Sir Sunderlal Hospital, Banaras Hindu University. Patients with hepatic or metabolic comorbidities were excluded. Clinical characteristics and hematological and biochemical parameters were recorded. Resected gallbladder specimens were examined for microanatomical and histological alterations. Statistical analysis was performed using IBM Corp. Released 2026. IBM SPSS Statistics for Windows, Version 26. Armonk, NY: IBM Corp., with p<0.05 considered significant.

Results: A total of 100 patients with cholelithiasis were stratified into four clinical severity grades. Of these, 36 were male, and 64 were female. The mean age showed no significant variation across the groups (p = 0.480), indicating a uniform age distribution among the study population. Serum alkaline phosphatase levels showed a marked, statistically significant rise with increasing severity (p < 0.0001). Significant inter-group differences were also observed in hemoglobin, platelet count, bilirubin fractions, total protein, urea, and selected anthropometric parameters (p < 0.05). Histopathological evaluation revealed epithelial hyperplasia as the most common lesion (58%), followed by low-grade dysplasia (22%) and intestinal metaplasia (10%), while normal mucosa was seen in only 10% of cases (p < 0.001). These findings demonstrate a clear progression of microanatomical alterations consistent with the metaplasia-dysplasia-carcinoma sequence.

Conclusion: Chronic cholelithiasis was associated with significant mucosal changes, mainly epithelial hyperplasia, intestinal metaplasia, and low-grade dysplasia, supporting the metaplasia-dysplasia-carcinoma sequence. Alkaline phosphatase levels rose progressively with disease severity, indicating ongoing tissue injury. These findings highlight the importance of routine histopathological examination for early detection of precursor lesions.

## Introduction

Cancer remains a major global health concern, accounting for nearly 20 million new cases and 10 million deaths in 2022 [1]. A 77% rise in global cancer incidence is projected by 2050, with rates expected to increase from 529.40 (95% CI: 525.41-533.38) per 100,000 in 2022 to 549.17 (95% CI: 487.43-610.92) per 100,000 by 2031 [1]. India is predicted to experience a substantial rise in cancer incidence and mortality, underscoring the need for improved preventive and diagnostic strategies [2].

Cancer distribution varies widely due to differences in genetic background, socioeconomic status, lifestyle, and environmental exposures. Northern and Central India show particularly high and rising cancer burdens, especially among women [3,4]. Gallbladder cancer (GBC) reflects this regional variability, with high to moderate incidence reported in India, South America, East Asia, and Central Europe [5,6]. Gallbladder adenocarcinoma is an aggressive malignancy with outcomes closely linked to stage at diagnosis, ranging from 80% five-year survival in carcinoma in situ to 2% in advanced stage IVb disease [7,8]. Although laparoscopic cholecystectomy has increased the detection of incidental GBC, surgical spillage may lead to peritoneal dissemination, highlighting the need for reliable preoperative markers [9].

Gallbladder carcinogenesis is traditionally described through the adenoma-carcinoma and metaplasia-dysplasia-carcinoma pathways. However, the diverse mucosal alterations associated with cholelithiasis suggest additional mechanisms. Cholelithiasis is highly prevalent worldwide, although its incidence varies considerably across regions. In India, surgical data estimate a prevalence of around 4%, compared with 10-20% reported in many Western populations [10]. While most individuals with gallstones remain asymptomatic, a subset gradually develops symptoms over time. Gallstones induce a spectrum of histopathological changes, including inflammation, hyperplasia, cholesterolosis, metaplasia, dysplasia, and ultimately carcinoma, changes that may relate to gallstone characteristics such as their number, size, and morphology. The presence of gallstones was associated with a markedly increased risk of biliary tract cancers overall (OR 4.38; 95% CI 3.23-5.93), with particularly strong associations for gallbladder cancer (OR 7.26; 95% CI 4.33-12.18), extrahepatic bile duct cancer (OR 3.17; 95% CI 2.24-4.50), and ampulla of Vater cancer (OR 3.28; 95% CI 1.33-8.11) [11].

Understanding gallstone-related mucosal responses is essential for identifying precancerous lesions and guiding early surgical intervention, particularly in high-risk regions. This study aims to characterize precancerous gallbladder lesions in cholelithiasis and assess their histopathological features and clinical relevance in early detection and cancer prevention.

## Materials and methods

This cross-sectional observational study was conducted in the Department of Anatomy and General Surgery at Sir Sunderlal Hospital, Institute of Medical Sciences, Banaras Hindu University (BHU), Varanasi, over a four-year period from July 2021 to June 2025. The study protocol received approval from the Institutional Ethics Committee of the Institute of Medical Sciences, BHU (Approval No. Dean/2021/EC/2999), and written informed consent was obtained from all participants before enrollment. All procedures adhered to the ethical standards of the institution and the Declaration of Helsinki.

Sample size calculation

The sample size was calculated using G*Power software (Version 3.1.9.7; Heinrich-Heine-Universität, Düsseldorf, Germany). Based on data from previous Indian and international studies reporting a 15-30% prevalence of premalignant lesions in cholelithiasis, an estimated effect size of 0.30 was adopted [10]. With a power of 80%, an alpha level of 0.05, and a two-tailed analysis, the minimum required sample size was approximately 92 patients. To improve statistical robustness, compensate for possible exclusions, and enhance the representativeness of histopathological patterns, the sample size was rounded to a final cohort of 100 patients.

Inclusion criteria

Patients were eligible for inclusion if they were 18 years of age or older, either male or female, presented with right upper quadrant abdominal pain, and had a definitive diagnosis of cholelithiasis established on ultrasonography. Only individuals scheduled for elective laparoscopic or open cholecystectomy and willing to provide written informed consent were enrolled in the study.

Exclusion criteria

Patients were excluded if there was any clinical or radiological suspicion of gallbladder malignancy before surgery. Individuals with systemic or metabolic disorders known to influence gallbladder pathology, including diabetes mellitus, thyroid dysfunction, hyperlipidemia, fatty liver disease, and hepatic cirrhosis, were not considered for participation. Additional exclusion criteria comprised acute systemic illnesses at presentation, a history of prior biliary surgery or intervention, and cases with incomplete clinical, biochemical, or histopathological records that could compromise data integrity and analysis.

Clinical severity grading

Patients were assigned to four clinical grades based on presenting symptoms and mode of admission: Grade 1: Incidental gallbladder findings without biliary symptoms; Grade 2: Mild to moderate right hypochondrial pain without systemic features; Grade 3: Moderate to severe pain with clinical features suggestive of acute biliary colic; Grade 4: Emergency admissions with severe, intolerable pain or complications (e.g., suspected acute cholecystitis). Grading decisions were made by the operating surgeon at the time of admission and recorded prospectively [12].

Clinical, biochemical, and histopathological evaluation

A detailed clinical assessment was performed for all eligible patients, including demographic information, presenting symptoms, and relevant medical history. Preoperative hematological and biochemical parameters, such as hemoglobin, leukocyte indices, platelet count, liver function tests, renal markers, and serum electrolytes, were recorded. Following cholecystectomy, gallbladder specimens underwent a systematic gross examination to evaluate wall thickness, mucosal appearance, presence of stones, and any focal abnormalities. Representative tissue samples were taken from the fundus, body, and neck, as well as from any macroscopically suspicious areas. The tissues were fixed in 10% neutral buffered formalin, routinely processed, embedded in paraffin, and sectioned at 4-5 µm thickness. Hematoxylin and eosin (H&E)-stained sections were examined under a light microscope by experienced histopathologists, and all observed microanatomical and histological alterations, including epithelial hyperplasia, metaplasia, dysplasia, and inflammatory changes, were documented.

Statistical analysis

All data were first compiled in Microsoft Excel (Redmond, USA) and subsequently analyzed using statistical analysis performed using IBM Corp. Released 2026. IBM SPSS Statistics for Windows, Version 26. Armonk, NY: IBM Corp. The normal distribution of continuous variables was tested by using the Shapiro-Wilk test, and homogeneity of variances was evaluated using Levene’s test. When the assumptions of normality or equal variance were violated, appropriate nonparametric tests (Mann-Whitney U test or Kruskal-Wallis test) were employed. Descriptive statistics were used to summarize the baseline characteristics. Categorical variables were compared using the chi-square test, while continuous variables were analyzed using the independent samples t-test or one-way analysis of variance (ANOVA), depending on suitability. A p-value of <0.05 was considered to indicate statistical significance.

## Results

A total of 100 patients with cholelithiasis were evaluated and categorized into four groups based on the severity of their clinical condition (Table 1). Based on the clinical grading of patients with cholelithiasis, alkaline phosphatase (ALP) levels showed an upward trend with increasing disease severity. Grade 1 patients had the lowest ALP values (64.60±18.62 IU/L), which increased sharply in Grade 2 (209.16±29.02 IU/L) and Grade 3 (284.31±12.95 IU/L). The highest values were observed in Grade 4, with a mean ALP of 337.14±32.67 IU/L. A one-way ANOVA demonstrated a highly significant difference in ALP levels across the four clinical severity grades (F = 399.79, p < 0.0001) (Table 1).

**Table 1:** Grading of cholelithiasis severity with corresponding clinical features and alkaline phosphatase (ALP) levels. ALP: Alkaline Phosphatase; ALP values were measured in IU/L; SD: Standard Deviation; p-value calculated using one-way ANOVA; * Statistically Significant.

Category	Grade	Clinical Description	N (100)	ALP Levels			
				Mean ± SD (IU/L)	Range (IU/L)	F-Value	p-Value
1	Grade 1	Patients admitted for other reasons; incidental findings of gallbladder wall thickening/swelling; no direct signs or symptoms of cholelithiasis.	20	64.60±18.62	44-150	367.35	< 0.0001*
2	Grade 2	Mild to moderate pain in the right hypochondrium; discomfort present.	50	209.16±29.02	151-250		
3	Grade 3	Mild to severe pain in the right hypochondrium.	16	284.31±12.95	251-300		
4	Grade 4	Severe, intolerable pain; patients rushed to the hospital and were admitted through the emergency department.	14	337.14±32.67	> 300		

Demographic analysis showed that among 100 participants, 36 were males and 64 were females; their age did not differ significantly among the groups (p = 0.480), indicating uniformity in age distribution. The mean height of participants was 159.62±10.63 cm, with a range of 140-197 cm. The mean body weight was 64.66±12.25 kg, ranging between 40 and 103 kg. The BMI (kg/m²) index demonstrated a mean value of 24.37±2.88, with a minimum of 16.90 and a maximum of 32.00. However, height (p=0.043), weight (p=0.020), and BMI (p=0.025) showed statistically significant variation among different groups. Group 4 patients, those with the most severe symptoms, had notably lower mean height (153.50±6.64 cm), weight (56.36±10.75 kg), and BMI (22.48±2.72 kg/m²) compared to the other groups. These findings suggest that anthropometric characteristics differed significantly across severity grades, even though age remained comparable (Table 2). Biochemical parameters revealed several statistically significant variations (Table 3). Hemoglobin levels decreased significantly with increasing severity (p = 0.003), reaching the lowest value in Group 4 (10.59±1.75 g/dL). Platelet counts also showed a significant difference across groups (p<0.001), with Group 1 showing higher platelet counts than the more symptomatic groups.

**Table 2:** Comparison of demographic parameters among the four study groups. SD: Standard Deviation; BMI: Body mass index; p-values calculated using one-way ANOVA; * Statistically Significant.

Variable	Group 1 (n=20) Mean±SD	Group 2 (n=50) Mean±SD	Group 3 (n=16) Mean±SD	Group 4 (n=14) Mean±SD	F-Value	p-Value
Age (years)	42.90±15.83	39.50±13.93	46.06±13.65	40.50±16.78	0.90	0.480
Height (cm)	160.95±9.72	161.42±11.94	157.69±8.39	153.50±6.64	2.40	0.043*
Weight (kg)	67.95±9.98	65.10±13.16	66.44±10.77	56.36±10.75	2.92	0.020*
BMI (kg/m²)	24.96±3.19	24.35±2.58	25.34±2.99	22.48±2.72	3.07	0.025*

**Table 3:** Comparison of biochemical parameters among the four study groups. SD: Standard Deviation; TLC: Total Leukocyte Count; TSH: Thyroid-Stimulating Hormone; SGOT: serum glutamic-oxaloacetic transaminase), SGPT: serum glutamate pyruvate transaminase, p-values calculated using one-way ANOVA; * Statistically Significant.

Variable	Group 1 (n=20) Mean±SD	Group 2 (n=50) Mean±SD	Group 3 (n=16) Mean±SD	Group 4 (n=14) Mean±SD	F-Value	P-Value
Hemoglobin (g/dl)	12.14±1.54	12.28±1.95	11.86±1.55	10.59±1.75	3.35	0.003*
TLC	8646.50±2709.67	8801.60±2703.57	9521.88±3382.92	9945.00±3896.12	0.78	0.350
DLC1	64.43±17.59	71.39±12.77	62.76±12.46	62.50±12.44	2.17	0.435
DLC2	25.31±18.85	32.00±10.06	28.52±11.16	27.84±11.47	1.00	0.459
DLC1/DLC2	5.77±7.17	3.49±4.30	3.50±4.47	3.35±3.34	1.17	0.441
Platelets (×10³/µL)	276.9 ± 105.4	205.80±69.76	169.69±58.34	196.86±84.29	6.36	<0.001*
RBS	113.02±22.56	108.13±20.53	119.92±30.93	110.61±33.19	0.86	0.343
SGPT (U/L)	34.89±18.10	43.42±26.25	44.85±32.21	51.03±39.39	1.01	0.135
SGOT (U/L)	33.87±16.99	35.57±23.02	36.89±21.90	32.29±8.29	0.13	0.840
SGPT/SGOT	1.06±0.33	1.28±0.61	1.17±0.27	1.52±0.93	1.85	0.103
Total Bilirubin (mg/dL)	0.8±0.20	0.9±0.30	1.1±0.30	1.3±0.4	9.74	<0.0001*
Direct Bilirubin (mg/dL)	0.20±0.06	0.25±0.07	0.30±0.08	0.35±0.10	12.91	<0.0001*
TB/DB ratio	4.0±0.60	3.6±0.50	3.7±0.50	3.7±0.60	2.66	0.233
Total Protein (g/dL)	7.31±0.79	8.12±0.59	8.42±0.54	8.27±0.99	9.74	0.032*
Albumin (g/dL)	4.18±0.37	4.66±0.81	4.32±0.30	4.11±0.63	4.28	0.215
Creatinine (mg/dL)	0.82±0.20	0.82±0.20	0.83±0.20	1.18±0.25	11.97	0.065
Urea (mg/dL)	21.28±6.25	19.88±5.73	25.61±13.84	21.54±8.02	2.11	0.038*
Sodium (mmol/L)	137.32±3.40	138.93±2.91	138.80±3.30	137.1 ± 3.4	2.13	0.133
Potassium (mmol/L)	4.24±0.34	4.36±0.51	4.41±0.35	4.61±0.57	1.78	0.135
Chloride (mmol/L)	103.24±3.38	104.58±3.07	104.81±4.38	103.7 ± 3.7	0.98	0.271
Free T3 (pg/mL)	3.1 ± 0.4	3.2 ± 0.4	3.0 ± 0.5	3.1 ± 0.4	1.04	0.073
T4 (µg/dL)	7.8 ± 1.0	8.1 ± 1.1	8.3 ± 1.2	8.0 ± 1.1	0.67	0.113
TSH (µIU/mL)	2.55±0.78	3.37±2.41	2.75±1.18	3.31±1.94	1.07	0.375

Total bilirubin and direct bilirubin showed significant inter-group differences (p < 0.0001), whereas the TB/DB ratio did not vary significantly across severity grades (p = 0.233). Total protein levels were significantly different among the groups (p = 0.032), increasing from 7.31 ± 0.79 g/dL in Group 1 to 8.42 ± 0.54 g/dL in Group 3. Albumin levels were within normal physiological limits across all groups. Thyroid function tests remained normal in all severity categories; free T3 was 3.1 ± 0.4 pg/mL, and total T4 was 7.8 ± 1.0 µg/dL, with no significant inter-group variation observed. Electrolyte analysis showed no significant reduction in sodium or chloride levels across groups. Urea differed significantly (p = 0.038), with Group 3 showing the highest mean value (25.61 ± 13.84 mg/dL).

Other parameters, including TLC, differential leukocyte counts, random blood sugar (RBS), serum glutamic-oxaloacetic transaminase (SGOT), serum glutamate pyruvate transaminase) (SGPT), albumin, potassium, creatinine, and thyroid profile, did not show statistically significant differences (p > 0.05), indicating that these variables remained relatively stable across the severity spectrum.

Histopathological findings

Epithelial hyperplasia was the most frequent histological alteration, followed by low-grade dysplasia and intestinal metaplasia, while normal mucosa was present in only a small proportion of cases. The overall distribution of lesions was statistically significant (p < 0.001) (Table 4).

**Table 4:** Histomorphological spectrum of gallbladder mucosal changes. p-value calculated using Pearson’s chi-square test; * Statistically significant.

Sr. No.	Lesion type	No. of Cases (n = 100)	Percentage (%)	Chi-square (χ²) Value	p-Value
1.	Normal Gallbladder Epithelium	10	10%	61.92	<0.001*
2.	Epithelial Hyperplasia	58	58%		
3.	Low-Grade Dysplasia	22	22%		
4.	Intestinal Metaplasia	10	10%		
Total		100	100%		

Normal gallbladder epithelium

In 10 cases involving patients admitted for unrelated conditions, the gallbladder mucosa was lined by a single layer of tall columnar epithelial cells with basally located, uniform nuclei and abundant clear cytoplasm. The epithelial surface displayed luminal folds without evidence of architectural distortion or nuclear atypia. The lamina propria consisted of loose connective tissue containing scattered lymphocytes and occasional blood vessels. The muscularis propria comprised irregularly arranged smooth muscle bundles. No features of dysplasia, metaplasia, or inflammation were identified.

Epithelial hyperplasia

In 58 cases, the mucosal lining exhibited marked hyperplastic changes characterized by epithelial stratification, hyperchromatic nuclei, moderate nuclear pleomorphism, vesicular chromatin, and prominent nucleoli. The stroma demonstrated thickened muscularis fibers, elongated and congested blood vessels, and a conspicuous lymphocytic infiltrate. These changes are consistent with epithelial hyperplasia associated with stromal remodeling and chronic inflammation.

Low-grade dysplasia

In 22 cases, histological sections showed hyperplastic glandular epithelium with hyperchromatic nuclei, vesicular chromatin, and irregular nuclear contours. The underlying stroma revealed areas of intestinal metaplasia and focal inflammatory cell infiltration. These findings indicate epithelial hyperplasia with metaplastic transformation and localized chronic inflammatory changes.

Intestinal metaplasia

**Figure 1:**
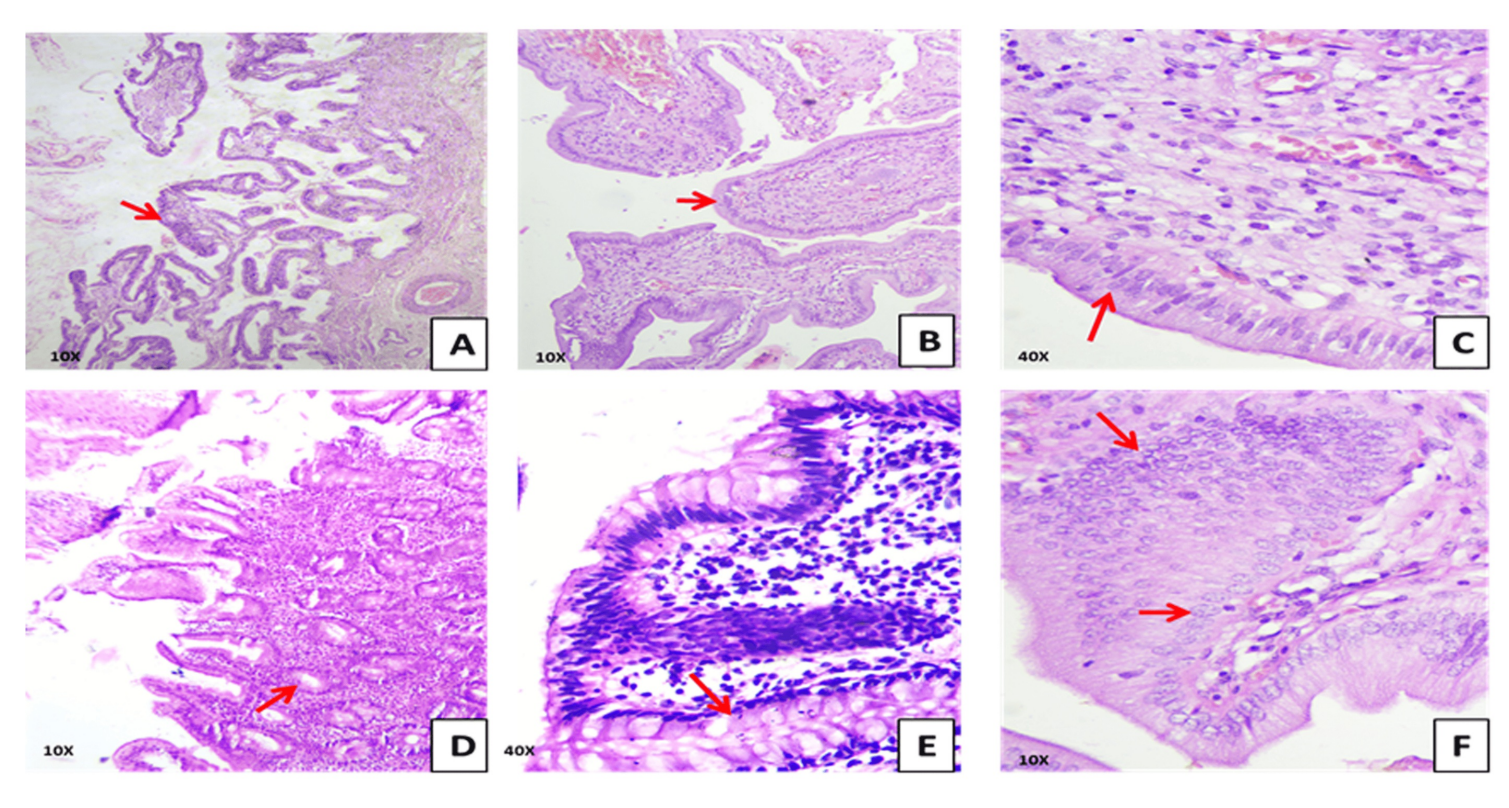
Histopathological spectrum of gallbladder mucosal alterations (H&E stain). Panel A shows papillary hyperplasia with complex mucosal folds at 10× magnification, while Panel B demonstrates focal epithelial proliferation with preserved architecture at the same magnification. Panel C depicts intestinal metaplasia characterized by columnar cells with basally located nuclei at 40×. Panel D illustrates marked mucosal hyperplasia with elongated and branched folds at 10×. Panel E shows goblet cell change consistent with intestinal metaplasia at 40×. Panel F presents low-grade dysplasia with nuclear crowding and stratification at 10×. Red arrows indicate representative lesions in each panel.

In 10 cases, normal gastric-type epithelium was replaced by intestinal-type mucosa. The epithelium consisted of tall columnar absorptive cells with basally placed nuclei interspersed with goblet cells containing intracytoplasmic mucin vacuoles. The nuclei demonstrated mild hyperchromasia with preserved polarity. The lamina propria contained focal infiltrates of lymphocytes and plasma cells. These features are consistent with intestinal metaplasia (Figure 1).

## Discussion

In this study, we evaluated hematological and biochemical parameters along with histopathological findings in 100 patients who underwent cholecystectomy for cholelithiasis. The majority of patients were female, consistent with the well-established higher prevalence of gallstone disease among women due to hormonal and metabolic factors [13-16].

Alkaline phosphatase (ALP) and disease severity

Our findings highlight the clinical utility of serum alkaline phosphatase (ALP) as a diagnostic marker in patients with cholelithiasis. We observed that higher ALP levels correlated with disease severity and could be stratified across different pain grades, supporting its role in risk assessment. These results are consistent with Costa et al. [17], who demonstrated in a cohort of 104 patients that an ALP cut-off of 78 U/L achieved 97.6% sensitivity and 72.6% specificity for detecting choledocholithiasis, with logistic regression identifying both age and ALP as significant predictors. Taken together, these findings reinforce the value of integrating ALP into preoperative diagnostic algorithms, potentially reducing dependence on costly imaging techniques such as magnetic resonance cholangiopancreatography (MRCP) while maintaining diagnostic accuracy. In line with this, Alessa et al. reported that patients with acute cholecystitis exhibited significantly elevated ALT and ALP levels compared to controls, highlighting the role of liver enzymes as predictors of biliary pathology. Their findings demonstrated that ALT and ALP, in combination with clinical diagnostic tools such as the Tokyo guidelines, improved diagnostic accuracy and patient management outcomes [18]. Complementing these findings, they showed that elevated liver enzymes in acute cholecystitis patients without choledocholithiasis were correlated with fatty liver and greater severity of radiologic findings, suggesting that abnormal liver profiles may not solely reflect ductal obstruction but can also indicate underlying hepatic pathology or disease burden [19]. Taken together, these studies support the role of ALP and other liver enzymes as accessible, cost-effective, and clinically relevant biomarkers that, when interpreted in the appropriate clinical context, can enhance diagnostic precision, guide preoperative decision-making, and potentially reduce reliance on expensive imaging modalities such as MRCP.

Histopathological correlation

Our study demonstrates a spectrum of histological changes in gallbladder mucosa among patients undergoing cholecystectomy. Epithelial hyperplasia was the most common alteration (58%), followed by low-grade dysplasia (22%) and intestinal metaplasia (10%), with normal epithelium preserved in only 10% cases. These findings highlight the chronic inflammatory and metaplastic responses of the gallbladder epithelium to calculous irritation and reinforce the concept of a stepwise progression from inflammation to neoplastic transformation. Hyperplasia represents an adaptive response to persistent mucosal injury and has been identified as a precursor lesion in the multistep sequence of gallbladder carcinogenesis [20-22].

Influence of gallstone characteristics and symptom duration

The relationship between gallstones and histological changes has been extensively studied. Singh et al. reported that gallstone size, rather than type or number, significantly influenced mucosal transformation, progressing from cholecystitis to hyperplasia, metaplasia, and carcinoma [23]. Sharma et al. observed that although age, stone size, and number were initially associated with carcinoma in univariate analysis, only the duration of symptoms remained significant in multivariate analysis [24]. This suggests that while gallstone characteristics contribute to mucosal injury, the chronicity of disease is likely the dominant factor driving premalignant and malignant transformation. Our findings of advanced mucosal changes in longstanding cholelithiasis cases are consistent with these observations.

Preinvasive neoplasms and risk factors

The recognition of defined preinvasive lesions provides further insight into gallbladder carcinogenesis. Fukumura described pyloric gland adenoma (PGA), biliary intraepithelial neoplasia (BilIN), and intracholecystic papillary neoplasm (ICPN) as the major preinvasive neoplasms, with additional subtypes such as intracholecystic papillary-tubular neoplasm and intracholecystic tubular non-mucinous neoplasm (ICTN) [25]. Chronic calculous irritation, anatomical variations like the pancreatobiliary junction (PBM), and prolonged inflammatory insults converge on these neoplastic pathways [22]. The importance of large gallstones, chronic infection, and gallbladder polyps as significant risk factors for carcinoma emphasizes the multifactorial etiology of gallbladder malignancy [26].

Biochemical correlations and clinical relevance

Biochemical markers such as liver enzymes offer complementary diagnostic information. In our study, elevated alkaline phosphatase (ALP) correlated with disease severity and pain grades. Costa et al. demonstrated that ALP is a sensitive predictor of choledocholithiasis, while Alessa et al. and Hee confirmed that elevations in ALP and ALT are associated with more severe histological and radiological findings [17-19]. Together, these findings suggest that integrating histopathology with biochemical markers can improve preoperative risk stratification and guide early intervention strategies.

Clinical implications

The combined evaluation of hematological, biochemical, and histological parameters provides a comprehensive understanding of disease progression in cholelithiasis. Elevated ALKP, in particular, may serve as a useful marker for identifying patients at risk of severe histological damage and operative complications.

Limitations of the study

This study was conducted at a single center with a relatively small sample size and excluded patients with systemic metabolic disorders, which may limit the generalizability of the findings to broader cholelithiasis populations. The absence of a control group without gallstones further restricts the ability to compare baseline mucosal changes. As a cross-sectional analysis, the study could not evaluate the temporal progression of precursor lesions. Additionally, molecular markers such as p53 or Ki-67, as well as advanced imaging modalities, were not included in the analysis. This was primarily due to resource constraints and the specific objective of focusing on routine H&E-based histopathology rather than molecular characterization. Incorporating these techniques could have provided deeper insights into early carcinogenic pathways. Future studies with larger, multicentric cohorts and integrated molecular and imaging assessments are needed to validate and expand upon these observations.

## Conclusions

Our findings demonstrate that chronic cholelithiasis is associated with progressive mucosal alterations, most commonly epithelial hyperplasia, intestinal metaplasia, and low-grade dysplasia, supporting the established metaplasia-dysplasia-carcinoma sequence. The observed stepwise rise in alkaline phosphatase with increasing clinical severity further indicates ongoing epithelial injury. These results highlight the importance of routine histopathological evaluation of all cholecystectomy specimens to facilitate early detection of precursor lesions and minimize the risk of missed malignancies.
